# Pharmacovigilance-based drug repurposing: searching for putative drugs with hypohidrosis or anhidrosis adverse events for use against hyperhidrosis

**DOI:** 10.1186/s40001-023-01048-z

**Published:** 2023-02-24

**Authors:** Yi Liu, Yanguo Liu, Rongrong Fan, Nurmuhammat Kehriman, Xiaohong Zhang, Bin Zhao, Lin Huang

**Affiliations:** 1 Department of Pharmacy, Peking University People’s Hospital, Beijing, China; 2 Department of Thoracic Surgery, Peking University People’s Hospital, Beijing, China; 3 Department of Pharmaceutical Analysis, School of Pharmacy, Peking University, Beijing, China; 4 Department of Pharmacy, Peking Union Medical College Hospital, Peking Union Medical College, Chinese Academy of Medical Sciences, Beijing, China

**Keywords:** Hyperhidrosis, Pharmacovigilance, Drug repurposing, FAERS, Hypohidrosis, Anhidrosis

## Abstract

**Background:**

Drug repurposing refers to the application of existing drugs to new therapeutic indications. As phenotypic indicators of human drug response, drug side effects may provide direct signals and unique opportunities for drug repurposing.

**Objectives:**

We aimed to identify drugs frequently associated with hypohidrosis or anhidrosis adverse reactions (that is, the opposite condition of hyperhidrosis) from the pharmacovigilance database, which could be potential candidates as anti-hyperhidrosis treatment agents.

**Methods:**

In this observational, retrospective, pharmacovigilance study, adverse event reports of hypohidrosis or anhidrosis in the US Food and Drug Administration (FDA) Adverse Event Reporting System (FAERS) were assessed between January 2004 and December 2021 using reporting odds ratio (ROR) estimates and categorized by the World Health Organization Anatomical Therapeutic Chemical (ATC) classification code. The onset time of drug-associated hypohidrosis or anhidrosis was also examined.

**Results:**

There were 540 reports of 192 drugs with suspected drug-associated hypohidrosis or anhidrosis in the FAERS database, of which 39 drugs were found to have statistically significant signals. Nervous system drugs were most frequently reported (187 cases, 55.82%), followed by alimentary tract and metabolism drugs (35 cases, 10.45%), genitourinary system and sex hormones (28 cases, 8.36%), and dermatologicals (22 cases, 6.57%). The top 3 drug subclasses were antiepileptics, drugs for urinary frequency and incontinence, and antidepressants. Taking disproportionality signals, pharmacological characteristics of drugs and appropriate onset time into consideration, the main putative drugs for hyperhidrosis were glycopyrronium, solifenacin, oxybutynin, and botulinum toxin type A. Other drugs, such as topiramate, zonisamide, agalsidase beta, finasteride, metformin, lamotrigine, citalopram, ciprofloxacin, bupropion, duloxetine, aripiprazole, prednisolone, and risperidone need more investigation.

**Conclusions:**

Several candidate agents among hypohidrosis or anhidrosis-related drugs were identified that may be redirected for diminishing sweat production. There are affirmative data for some candidate drugs, and the remaining proposed candidate drugs without already known sweat reduction mechanisms of action should be further explored.

## Introduction

Drug repurposing is the process of identifying and developing novel disease indications for approved drugs [1]. The advantage of drug repurposing over conventional drug development is that it can reduce the time, cost, and risk of failure associated with new drug development [2]. Other than approaches that are primarily focused on omics- or ligand docking, adverse event (AE)-based drug repurposing is a novel strategy [3]. Each drug interacts with multiple pharmacodynamic targets, producing the expected therapeutic activity and undesired effects, which may be either beneficial or harmful AEs. These multitarget profiles explain how one drug might have therapeutic relevance in several diseases, especially when these diseases share common genes, biomarkers, signaling pathways, or environmental factors [4]. Thus, drug screening based on AEs could reveal unknown pharmacodynamic properties.

Pharmacovigilance data consist of spontaneously reported AEs, and several databases are available, such as the US Food and Drug Administration (FDA) Adverse Event Reporting System (FAERS), European Medicines Agency (EMA) EudraVigilance and World Health Organization (WHO) VigiBase [5]. Disproportionate analysis, a drug–event combination that occurs more frequently than expected, helps to estimate whether these AEs occurred randomly or were caused by the drug and can, therefore, be considered a true adverse drug reaction [6]. Pharmacovigilance data have been employed to generate hypotheses for new therapeutic applications of existing drugs [7–11]. For instance, it was used to detect which drugs were associated with significantly overreported hypotension and assess the opportunity to use them as antihypertensive agents based on the FAERS database [10]. Zamami Y et al. identified prophylactic drugs for oxaliplatin-induced peripheral neuropathy by drug repositioning using data from the FAERS database [11].

Hyperhidrosis is an idiopathic disorder characterized by excessive sweating not related to heat or physical exercise, which may have a significant negative impact on a person’s quality of life, causing both physical problems and psychological distress [12]. The prevalence rate of hyperhidrosis in the general population in the US was reported to be 4.8% [13]. Further estimates of its prevalence have varied widely, ranging from 4.6% in Germany to 5.5% in Sweden; 12.3% in Vancouver, Canada; and 14.5% in Shanghai, China [14–16]. A variety of nonsurgical and surgical treatments currently exist to manage hyperhidrosis. Nonsurgical treatments include topical therapies, oral anticholinergic medications, iontophoresis, and injectable medications. Aluminum chloride is the most commonly used and effective topical medication, but its satisfaction rate is only 60% [17]. Anticholinergic or astringent medications are topically used as well. However, the common disadvantage of all of these topical therapies is inadequate long-term efficacy [18]. Oral anticholinergic medications, such as glycopyrrolate or oxybutynin, have been used off-label for hyperhidrosis, with a response rate of approximately 70%, but one-third of patients discontinue treatment due to adverse effects [19]. Iontophoresis is another effective treatment for hyperhidrosis, with an effective rate of approximately 80%, but maintenance therapy must be given every 1–4 weeks [20]. Botulinum toxin is an injectable medication used for hyperhidrosis, but adverse effects, including pain and haematoma caused by injection, limit its usage. It is usually highly effective within the first 7–10 days after therapy, but only lasts for 3–9 months or longer and requires repeated injections to maintain its effects [21]. Surgical interventions, such as endoscopic thoracic sympathectomy, are usually reserved for severe cases in which other less invasive and more conservative measures have failed [17]. However, persistent side effects such as compensatory sweating and Horner’s syndrome may result from the surgery. To date, there are no available drugs or methods with stable and durable effects. Compared with other skin disorders of similar prevalence, hyperhidrosis is under researched [22]. Assuming that hypohidrosis or anhidrosis, with decreased sweating or complete absence of sweating, can be regarded as the opposite conditions of hyperhidrosis, potential anti-hyperhidrosis agents may be discovered among drugs that induce hypohidrosis or anhidrosis as side effects.

To the best of our knowledge, this drug discovery approach has not been applied to anti-hyperhidrosis drug repurposing. Therefore, the objectives of the present study were to describe the hypohidrosis or anhidrosis cases submitted to the FAERS database, evaluate the signals between drugs and hypohidrosis or anhidrosis using disproportionality analyses, and examine the onset time of hypohidrosis or anhidrosis events after drug exposure. We hoped this study would provide unique insight into pharmacovigilance-based anti-hyperhidrosis drug repurposing and furnish possible treatment options for hyperhidrosis through indication expansion.

## Methods

### Study design and data source

We performed a retrospective pharmacovigilance study using the FAERS database from January 2004 to December 2021. The FAERS is one of the most comprehensive sources of pharmacovigilance big data worldwide. Data from the FAERS are routinely used by national drug regulating authorities to identify potential safety signals. Compared with other international spontaneous reporting systems, FAERS has unique characteristics, including providing public access to raw data and allowing for the recognition of the different role codes for drugs, such as primary suspect, secondary suspect, interacting, and concomitant agents [23]. As the database is an anonymized open-access database, institutional review board approval and informed consent were not needed.

The data sets consisted of several data tables as follows: “DEMO” table for demographic and administrative information, “DRUG” table for drug information, “REAC” table for adverse events, “RPSR” table for report sources, “THER” table for therapy dates for reported drugs, “OUTC” table for patient outcome, and “INDI” table for indications for drug use. We first removed duplicate records by choosing the earliest FDA_DT when the CASEIDs were the same and selecting the higher PRIMARYID when the FDA_DT and CASEIDs were the same. We finally included 14,467,172 cases from the FAERS database for further analysis.

### Data mapping

We inspected the REAC files for the comprehensive Medical Dictionary for Regulatory Activities Terminology (MedDRA version 25.0), and preferred terms linked to hypohidrosis or anhidrosis were defined as follows: hypohidrosis (code: 10,021,013), hyphidrosis (code: 10,020,926), sweating decreased (code: 10,042,664), anhidrosis (code: 10,002,512) and anhydrosis (code: 10,002,513). As different roles can be chosen by the submitter for the reported drug, exposure evaluation considered only drugs with a “role code” of ‘Primary Suspect’. In the “DRUG” table, drugs might be documented in various forms, such as brand names, generic names, synonymous names, or abbreviations. DrugBank was used to standardize different drug names of the same drug into “generic names”.

### Descriptive analysis

Descriptive analysis was used in our study to illustrate the characteristics of the cases of hypohidrosis or anhidrosis, including sex and age, reporting area and reporters, annual case reports, and outcomes. The descriptive analyses were conducted by Microsoft Excel version 2019 (Microsoft Corporation, Redmond, Washington, USA).

### Detection of drugs associated with hypohidrosis or anhidrosis

Disproportionality analysis in pharmacovigilance databases is a validated method in drug surveillance research [24]. To explore the association between certain drugs and hypohidrosis or anhidrosis, we calculated the reporting odds ratio (ROR) as the ratio of the odds of reporting hypohidrosis or anhidrosis adverse events vs. all other events for a given drug to the reporting odds for all other drugs. The equations and criteria for the algorithms are shown in Table 1 [25]. Drugs identified were classified based on the drug name and Anatomical Therapeutic Chemical (ATC) codes.

**Table 1:** Algorithms used for signal detection

Algorithms	Equation	Criteria
ROR	ROR = (a/b)/(c/d)	95% CI >1, N ≥ 2
	95%CI = e^ln(ROR)±1.96(1/a+1/b+1/c+1/d)^0.5^	

a: number of reports containing both the suspect drug and the suspect adverse drug reaction; b: number of reports containing the suspect adverse drug reaction with other medications (except the drug of interest); c: number of reports containing the suspect drug with other adverse drug reactions (except the event of interest); d: number of reports containing other medications and other adverse drug reactions

*ROR* reporting odds ratio, *CI* confidence interval, *N* the number of co-occurrences

We estimated the onset time of hypohidrosis or anhidrosis after drug exposure, which was defined as the time interval between the EVENT_DT (onset date of hypohidrosis or anhidrosis) and the START_DT (start date of the drug). We excluded records with incorrect or erroneous inputs (START_DT later than EVENT_DT).

### Statistical analysis

As the data were not normally distributed with uneven variance, non-parametric tests (Dunn’s multiple comparison test for dichotomous variables and the Kruskal‒Wallis test for more than two subgroups of respondents) were used to compare the time to onset. The statistical significance was set at *p* < 0.05 with 95% CI. The statistical analyses were conducted by GraphPad Prism version 8.0.2 (GraphPad Software, San Diego, California, USA).

## Results

### Descriptive analysis

**Fig. 1:**
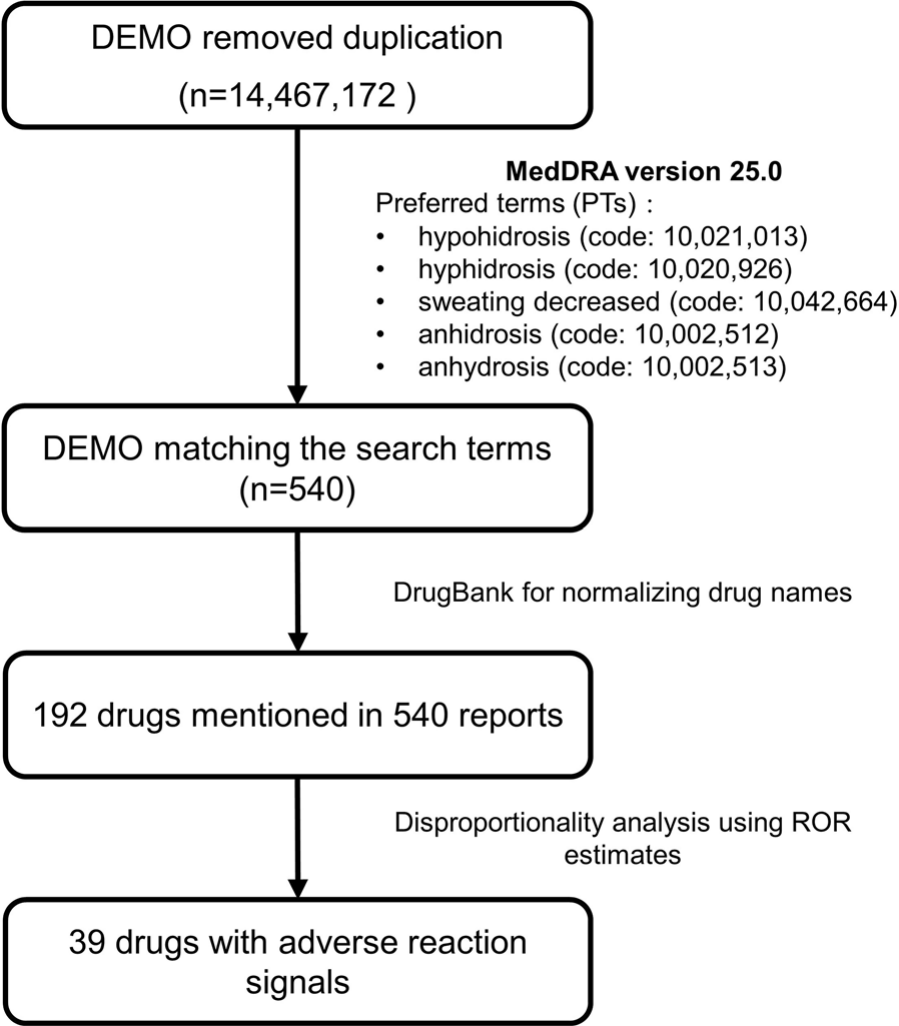
Process of the selection of cases of drug-associated hypohidrosis or anhidrosis from the FAERS database. *FAERS* Food and Drug Administration’s Adverse Event Reporting System

We screened 540 reports of 192 drugs with suspected drug-associated hypohidrosis or anhidrosis based on the ‘primary suspects’ role code (Fig. 1) and summarized the detailed information of the clinical characteristics in Table 2. The affected patients were more often females than males (48.70% *vs.* 42.41%). Patients were most frequently aged between 18 and 64 years (47.69%), with an average age of 38.90 ± 21.25 years. Notably, 74 patients (13.70%) aged  < 18 years were identified in our study. The cases identified in the FAERS were mainly submitted by consumers (46.11%) and were mostly from North America (65.19%), Europe (16.85%), and Asia (12.41%). The number of reports fluctuated over the years and was highest in 2018. This might be due to all 22 metformin-related cases reported in 2018. The reported cases experienced other serious events: important medical event (76.94%), hospitalization (37.82%), life-threatening events (14.25%), disability (13.21%), required intervention to prevent permanent impairment/damage (2.59%), death (2.33%), and congenital anomaly (0.52%). In addition, we also analysed the 213 reports submitted by healthcare professionals, including physicians, pharmacists and other health professionals. As shown in Table 2, the patients affected were more often females than males and were mostly 18–64 years (48.36%), and most of the cases were from North America (47.89%), Europe (23.94%), and Asia (21.60%).

**Table 2:** Demographic and clinical characteristics of hypohidrosis or anhidrosis cases collected from the FAERS database

Characteristics	Total reports, *N.* (%)	Reports by the healthcare professionals, *N.* (%)
	540	213
Gender		
F	263 (48.70)	96 (45.07)
M	229 (42.41)	90 (42.25)
Unknown	48 (8.89)	27 (12.68)
Age group (years)		
< 18	74 (13.70)	41 (19.25)
18–44	128 (23.70)	60 (28.17)
45–64	131 (24.26)	43 (20.19)
65–74	22 (4.07)	8 (3.76)
75–84	10 (1.85)	2 (0.94)
> 85	3 (0.56)	2 (0.94)
Unknown	172 (31.85)	57 (26.76)
Reporting Area		
Asia	67 (12.41)	46 (21.60)
Europe	91 (16.85)	51 (23.94)
North America	352 (65.19)	102 (47.89)
Oceania	2 (0.37)	–
South America	4 (0.74)	2 (0.94)
Unknown	24 (4.44)	12 (5.63)
Reporters		
Consumer	249 (46.11)	–
Lawyer	6 (1.11)	–
Other health professionals	99 (18.33)	99 (46.48)
Physician	98 (18.15)	98 (46.01)
Pharmacist	16 (2.96)	16 (7.51)
Unknown	72 (13.33)	–
Reporting Year		
2004	18 (3.33)	10 (4.69)
2005	11 (2.04)	5 (2.35)
2006	20 (3.70)	4 (1.88)
2007	9 (1.67)	3 (1.41)
2008	13 (2.41)	6 (2.82)
2009	17 (3.15)	6 (2.82)
2010	29 (5.37)	14 (6.57)
2011	14 (2.59)	3 (1.41)
2012	16 (2.96)	5 (2.35)
2013	30 (5.56)	12 (5.63)
2014	55 (10.19)	19 (8.92)
2015	30 (5.56)	13 (6.10)
2016	50 (9.26)	25 (11.74)
2017	36 (6.67)	17 (7.98)
2018	66 (12.22)	43 (20.19)
2019	36 (6.67)	11 (5.16)
2020	48 (8.89)	9 (4.23)
2021	39 (7.22)	7 (3.29)
Unknown	3 (0.56)	1 (0.47)
Outcome		
Congenital anomaly	2 (0.52)	–
Death	9 (2.33)	7 (4.00)
Disability	51 (13.21)	11 (6.29)
Hospitalization	146 (37.82)	75 (42.86)
Life-threatening	55 (14.25)	35 (20.00)
Other serious (important medical events)	297 (76.94)	134 (76.57)
Required intervention to prevent permanent impairment/damage	10 (2.59)	4 (2.29)

### Disproportionality analysis

**Fig. 2:**
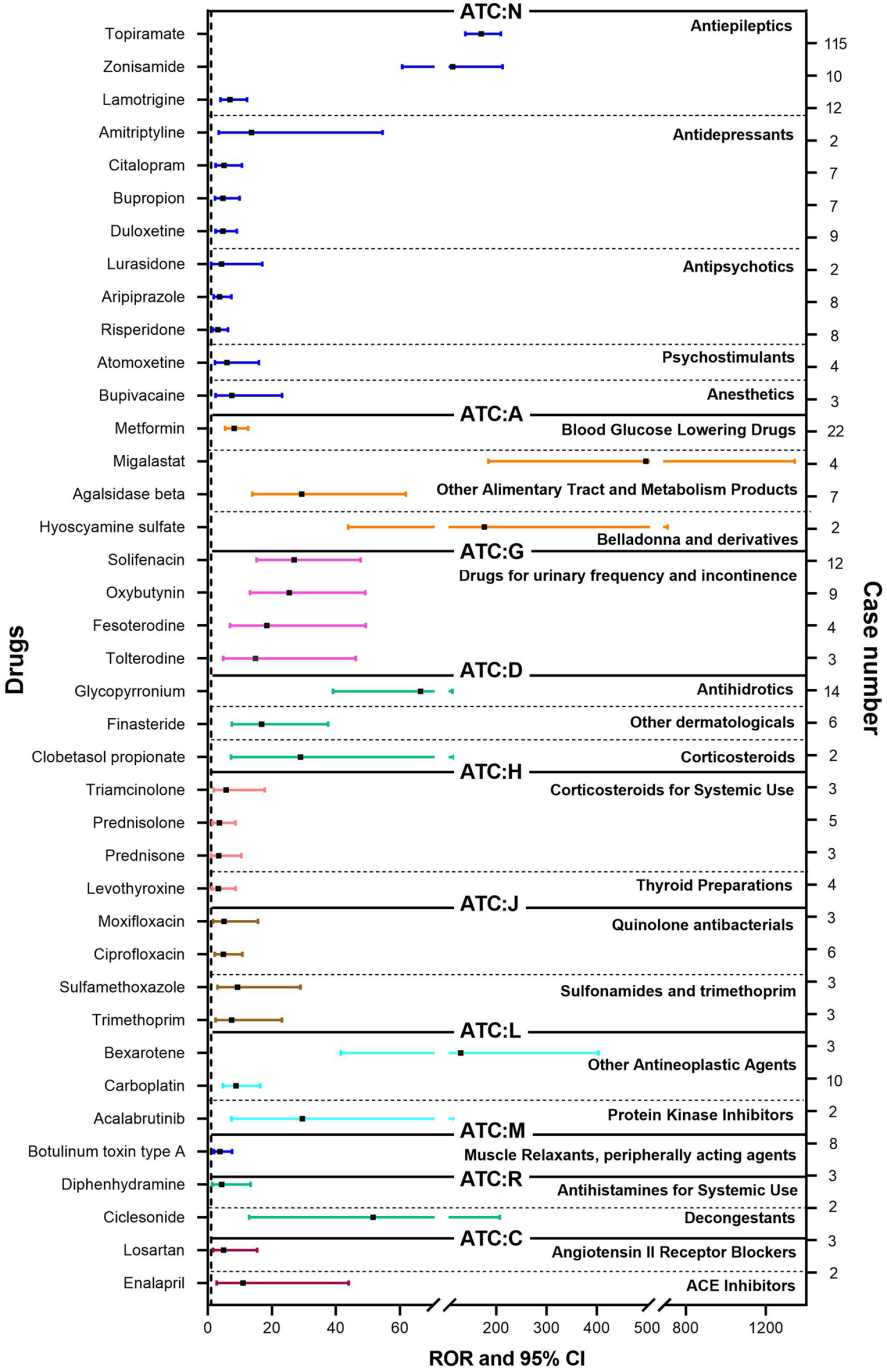
ROR and 95% two-sided CI of each drug associated hypohidrosis or anhidrosis in FAERS. *ROR* reporting odds ratio, *CI* confidence interval, *ATC:* *N* Nervous System Drugs, *ATC: A* Alimentary Tract and Metabolism Drugs, *ATC:* G Genitourinary System and Sex Hormones, *ATC:* *D* Dermatologicals, *ATC:* *H* Systemic Hormonal Preparations, Excl. Sex hormones and Insulins, *ATC: J* Anti-infectives for Systemic Use, *ATC:* *L* Antineoplastic and Immunomodulating Agents, *ATC:* *M* Musculo-Skeletal System, *ATC:* *R* Respiratory System Drugs, *ATC:* *C* Cardiovascular System Drugs

**Fig. 3:**
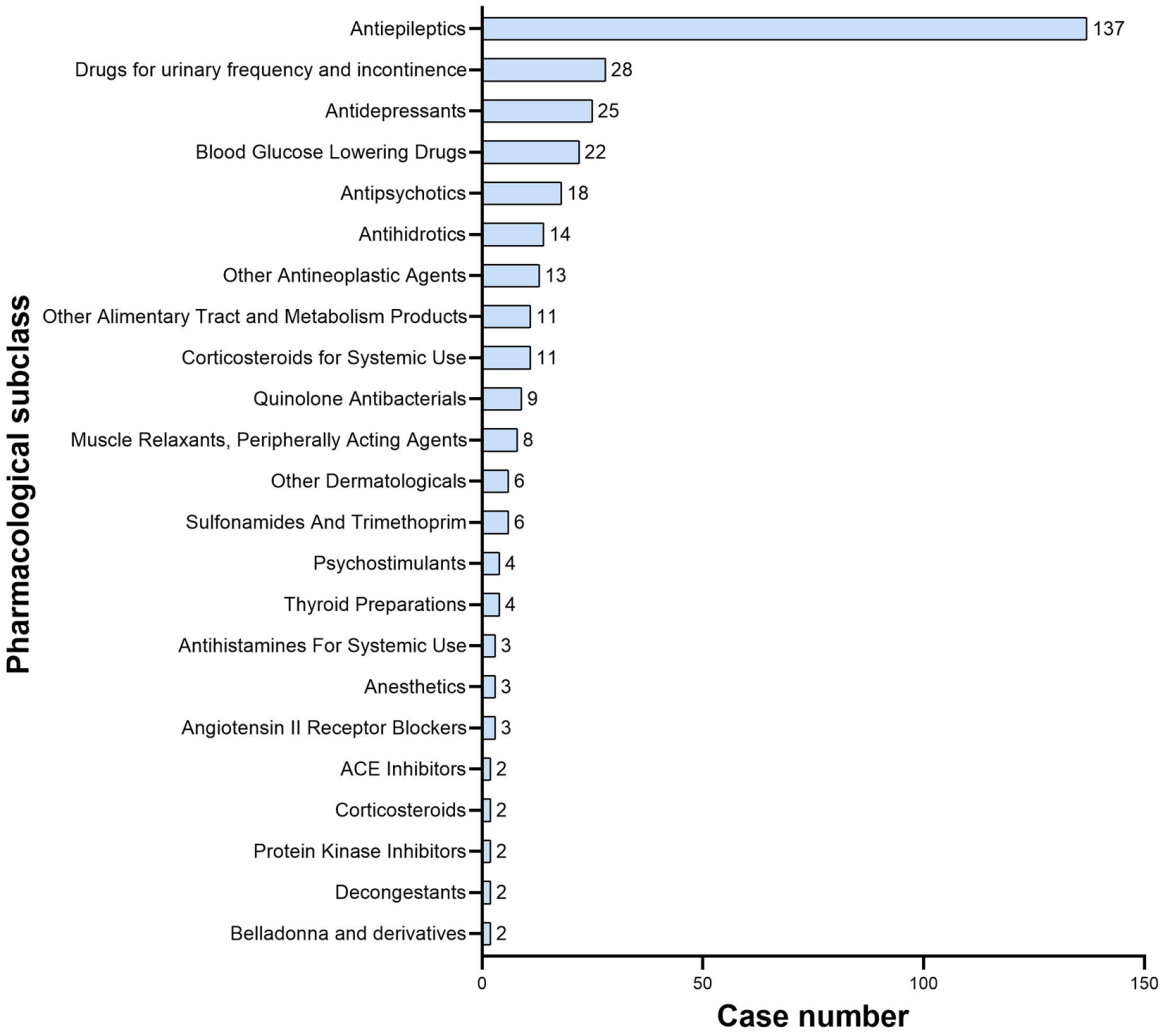
Reports of hypohidrosis or anhidrosis cases with different pharmacological subclasses

We detected hypohidrosis or anhidrosis signals for different drugs and recorded the results in Fig. 2. According to the standards of the algorithms, a total of 39 drugs fulfilled the search criteria. Among these, hypohidrosis or anhidrosis was recorded in the package insert for only 5 drugs (12.82%), including topiramate, zonisamide, hyoscyamine sulfate, oxybutynin, and glycopyrronium. Nervous system drugs (ATC:N) were most frequently reported (187 cases, 55.82%), followed by alimentary tract and metabolism drugs (ATC:A, 35 cases, 10.45%), genitourinary system and sex hormones (ATC:G, 28 cases, 8.36%) and dermatologicals (ATC:D, 22 cases, 6.57%). The top 3 drug subclasses associated with hypohidrosis or anhidrosis were antiepileptics (137 cases, 40.90%), drugs for urinary frequency and incontinence (28 cases, 8.36%), and antidepressants (25 cases, 7.46%), as shown in Fig. 3.

Moreover, to avoid false signals, 18 drugs were chosen based on  ≥ 5 reported cases. Topiramate was found to exhibit the highest ROR (ROR = 170.32, 95% two-sided CI 138.72–209.11), and the highest number of reported cases (115 cases), followed by zonisamide, glycopyrronium, agalsidase beta, solifenacin, oxybutynin, finasteride, carboplatin, metformin, lamotrigine, citalopram, ciprofloxacin, bupropion, duloxetine, botulinum toxin type A, aripiprazole, prednisolone, and risperidone. The drug list was further pruned: antineoplastic agents (e.g., carboplatin) were excluded because of their detrimental or unfavorable action on the patient. The removal of clinically infeasible drugs for the potential treatment of hyperhidrosis resulted in a list of 17 candidate drugs.

### Time to onset analysis

**Fig. 4:**
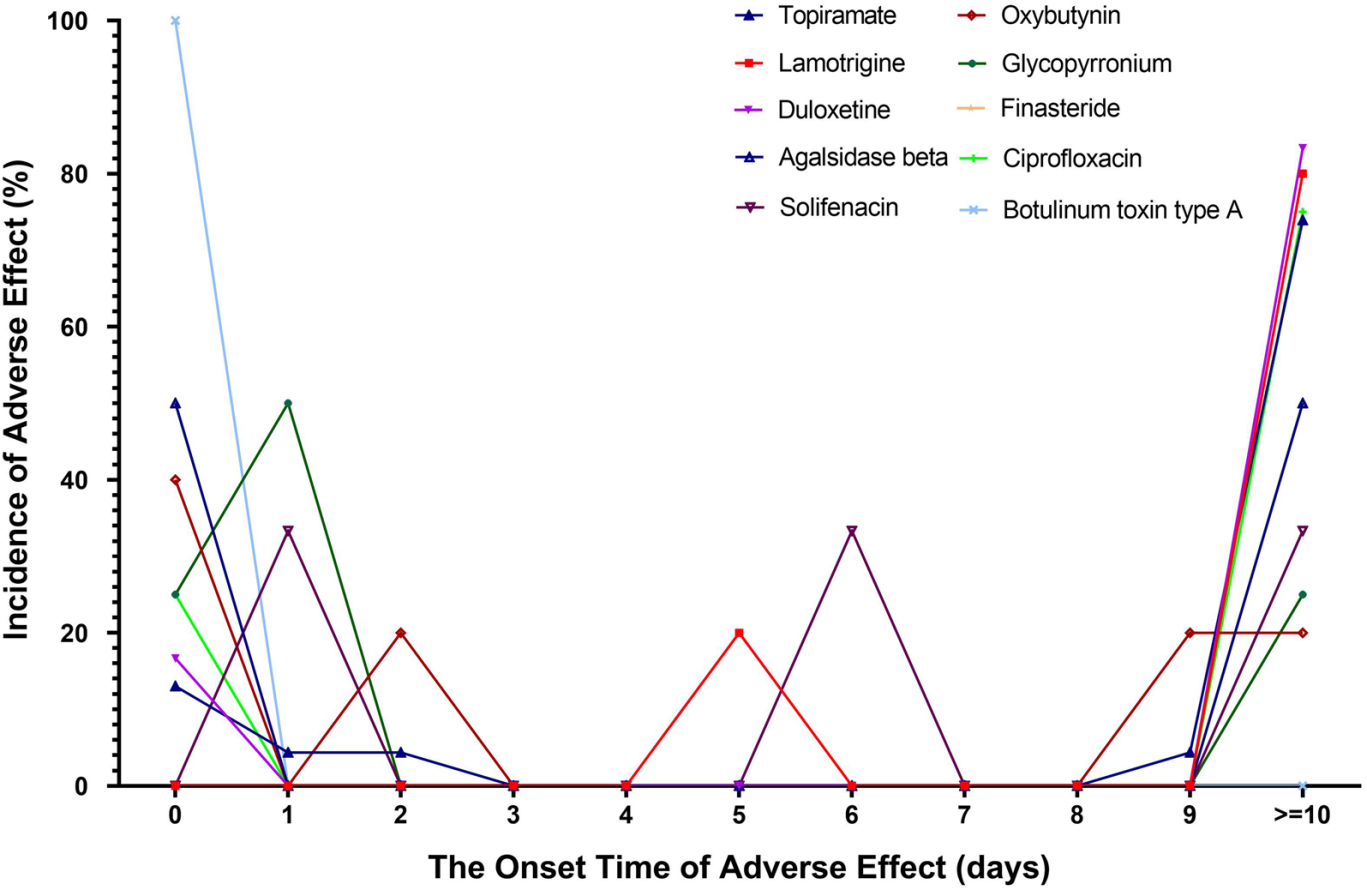
Time to event onset of hypohidrosis or anhidrosis following the candidate drugs exhibit the higher ROR and  ≥ 5 reported cases collected from the FAERS database. Drugs were not shown if the incidence of adverse effects (%) less than 10 days was 0% (zonisamide, citalopram, aripiprazole, and risperidone). Data was not available for bupropion, metformin and prednisolone

We estimated the time interval from drug exposure to hypohidrosis or anhidrosis onset among the 17 candidate drugs. Data were not available for bupropion, metformin, and prednisolone in the database. As shown in Fig. 4, the median onset times of botulinum toxin type A, glycopyrronium, oxybutynin, and solifenacin-associated hypohidrosis or anhidrosis were 0 days [interquartile range (IQR) 0–0], 1 day (IQR 0.75–17), 2 days (IQR 0–9) and 6 days (IQR 3.5–18.5), respectively, whereas, those of lamotrigine, agalsidase beta, topiramate, finasteride, duloxetine, and citalopram were 34 days (IQR 14–34), 53.5 days (IQR 26.75–80.25), 59 days (IQR 11.5–256), 68.5 days (IQR 34.25–102.75), 202 days (IQR 202–202), and 218.5 days (IQR 191.25–245.75), respectively. Zonisamide, citalopram, aripiprazole, and risperidone were not shown as the incidence of adverse effects (%) less than 10 days was 0%.

## Discussion

To the best of our knowledge, this is the first study using AE-based drug repurposing strategies to identify potential anti-hyperhidrosis agents. Based on pharmacovigilance analysis of real-world adverse events reported to FAERS, some putative drugs were found to have statistically significant signals with hypohidrosis or anhidrosis, which may be redirected for diminishing sweat production.

Our study first systematically assessed hypohidrosis or anhidrosis reports based on the FAERS database and is the largest summary of drug-associated hypohidrosis or anhidrosis cases to date. In our study, 540 reports of 192 drugs with suspected drug-associated hypohidrosis or anhidrosis from the FAERS database were assessed. Among them, 213 reports were submitted by healthcare professionals, including physicians, pharmacists and other health professionals. Detailed information on the clinical characteristics was summarized. Furthermore, 39 drugs were identified with hypohidrosis or anhidrosis signals according to disproportionality analysis. Nervous system drugs were most frequently reported, including antiepileptics, antidepressants and antipsychotics. The results were consistent with other previous studies [26]. A review demonstrated that the drugs with a higher correlation with hypohidrosis or anhidrosis were anticholinergics, tricyclic antidepressants, and antiepileptics [26], although evidence mainly came from case reports or a small sample population in a specific area [27–33].

To avoid false signals, 18 drugs were further chosen given  ≥ 5 reported cases [34]. In addition, the pharmacological characteristics of the drugs were also considered [35]. Carboplatin, which might be clinically infeasible, was removed from the candidate drug list. In addition to detecting hypohidrosis or anhidrosis signals, the time to onset of drug-induced hypohidrosis or anhidrosis was also evaluated, which would possibly be the time to onset of anti-hyperhidrosis therapy. Our results indicated that the median onset time of botulinum toxin type A, glycopyrronium, oxybutynin and solifenacin-associated hypohidrosis or anhidrosis was 0–6 days, whereas that of lamotrigine, agalsidase beta, topiramate, finasteride, ciprofloxacin, zonisamide, duloxetine, citalopram, risperidone and aripiprazole was 34–1210 days. If the interval between adverse reactions of hypohidrosis or anhidrosis after drug exposure is too long, it may indicate that the drug is inappropriate for the treatment of hyperhidrosis.

Considering disproportionality signals, pharmacological characteristics of drugs and appropriate onset time, our study suggests that the main putative drugs for anti-hyperhidrosis are glycopyrronium, solifenacin, oxybutynin, and botulinum toxin type A. Other drugs, such as topiramate, zonisamide, agalsidase beta, finasteride, metformin, lamotrigine, citalopram, ciprofloxacin, bupropion, duloxetine, aripiprazole, prednisolone, and risperidone need further investigation. This is in line with studies that used VigiBase to investigate drugs associated with hypohidrosis or anhidrosis and revealed that the most at-risk drugs are oxybutynin, topiramate, and zonisamide [36].

The spectrum of drugs highlights the various mechanisms of drug-related hypohidrosis or anhidrosis. Undoubtedly, understanding the components of the human thermoregulatory system can help classify the effects of drugs on sweating [37]. The sweating pathway begins in the medial preoptic area of the hypothalamus and extends to the intermediolateral column, from which preganglionic sympathetic fibers arise that leave the spinal cord and synapse within the sympathetic chain ganglia. Postganglionic sympathetic nerves emerge from these ganglia and travel alongside arteries to reach their subdermal targets, the eccrine sweat glands. Along this pathway, the most clinically important neuroendocrine mediator is acetylcholine, which binds to cholinergic muscarinic receptors in the basolateral membrane of the clear cell. Released acetylcholine leads to myoepithelial cell contraction, and sweat production is designated sudomotor activity [37–39]. In theory, drugs that interact with each level of this pathway can induce sweating disorder. These putative drugs for decreasing sweat production were categorized and discussed in the following pharmacological categories:

1. Antiepileptics, including topiramate, zonisamide and lamotrigine, can interfere with sweat production by inhibiting carbonic anhydrase, probably at the level of the secretory coil clear cell or apex of ductal cells. Hypohidrosis is well-described for antiepileptics both in adults and paediatrics [29, 40–42]. Studies have shown that paediatric patients taking zonisamide have an estimated tenfold higher risk of hypohidrosis than adults [43]. This means that the drug might work better in children than in adults when used to treat hyperhidrosis. However, the median onset time of hypohidrosis or anhidrosis after topiramate, zonisamide and lamotrigine exposure seems relatively long.
2. Among the antidepressants, citalopram was found to exhibit a higher ROR, followed by bupropion and duloxetine. These drugs might have the greatest potential to alter sweat production at the junction between the sympathetic nerve terminal and the eccrine sweat gland. Studies have identified correlations between the psychological characteristics of patients with hyperhidrosis [44]. Bahar et al. observed a higher prevalence of depression in patients with hyperhidrosis than in the general population and positive correlations between hyperhidrosis severity and the prevalence of anxiety and depression [45]. Therefore, if patients with hyperhidrosis are comorbid with depression, then antidepressants may be able to achieve dual purposes.
3. As one of the atypical antipsychotics, aripiprazole is increasingly prescribed together with antidepressants. Lu et al. presented two cases of antidepressant-induced sweating alleviated by aripiprazole. It was suggested that decreased sweating is linked to the unique property of aripiprazole as a partial dopamine agonist. Thus, aripiprazole may confer a homeostatic effect on the dopamine-dependent functions of the hypothalamus [46]. Huang et al. reported a case of developing hyperhidrosis under a combination of zotepine and haloperidol, in which the sweating subsided after switching from zotepine to aripiprazole [47]. However, no reports of hypohidrosis or anhidrosis caused by risperidone have been described in the literature.
4. A significant ROR was found for metformin which is approved for glycaemic control in patients with diabetes. Maudar V et al. described three patients with long-standing hot flashes, excessive sweating, and fatigue whose symptoms were ameliorated with metformin. It was suggested that metformin may have sympathoinhibitory actions that alleviate these symptoms [48]. As metformin has good safety profiles and pleiotropic effects, it is a good model for the new use of old drugs [49]. Future studies are needed to better explore its application in the treatment of hyperhidrosis.
5. Agalsidase beta, a recombinant human alpha-galactosidase A enzyme, is approved for intravenous treatment of Fabry disease. Fabry disease is a progressive, multisystemic, potentially life-threatening disorder caused by a deficiency of alpha-galactosidase A [50]. Our study indicates that agalsidase beta exhibits statistically significant hypohidrosis or anhidrosis signals. Paradoxically, hyperhidrosis is listed in its labeling in postmarketing surveillance. Further investigations are needed to interpret this distinction and possible mechanisms.
6. Our study demonstrated that both solifenacin and oxybutynin are the main putative drugs for anti-hyperhidrosis. Solifenacin and oxybutynin are anticholinergic medications that are indicated in patients with overactive bladder or symptoms of detrusor overactivity, including urinary frequency and urgency. Oxybutynin has been used for the off-label treatment of hyperhidrosis. Several studies have demonstrated that it is effective in both focal and generalized hyperhidrosis and shows a generally good response in different patients regardless of age, sex, and weight [51–54]. The most common adverse event is dry mouth reported by almost all treated patients [55]. However, there is no relevant report on solifenacin-induced hypohidrosis or anhidrosis.
7. Glycopyrronium, a topical anticholinergic agent, reduces sweat production by blocking the activation of acetylcholine receptors in peripheral sweat glands. Topical glycopyrronium tosylate, a pre-moistened cloth containing 2.4% glycopyrronium solution, was shown to be an effective, safe and non-invasive treatment for hyperhidrosis in clinical trials [56–59]. Topical glycopyrrolate is considered the first-line treatment for craniofacial sweating [60].
8. Botulinum toxin blocks the release of neuronal acetylcholine from the presynaptic junction of both neuromuscular and cholinergic autonomic neurons. By blocking the release of acetylcholine, botulinum toxin can temporarily reduce sweat production [61]. Botulinum toxin injections are used as a second-line hyperhidrosis treatment option once topical treatment strategies have failed [21].
9. A hypohidrosis or anhidrosis signal was found for prednisolone which is a synthetic adrenocortical steroid drug with predominantly glucocorticoid properties. However, the results were contrary to those reported in the literature. Yoritaka A et al. reported a case of acquired partial hypohidrosis with no underlying disease that was successfully treated with prednisolone 1000 mg/day for 3 days [62]. Kobayashi T et al. presented two cases of acquired idiopathic generalized anhidrosis that were successfully treated with methylprednisolone 1000 mg i.v. for 3 days [63]. They suggested that the therapy might have stimulated the functional recovery of the sweat glands by binding with unidentified receptors. Further studies are needed to explain this distinction.
10. No reports of hypohidrosis or anhidrosis caused by finasteride or ciprofloxacin have been described in the literature to date.

There are several limitations in our study. First, FAERS is a spontaneous reporting system with inherent disadvantages, including false reporting, under-reporting, and incomplete reporting, all of which may lead to reporting bias. Although the identity of the reporter is visible, inaccuracy and omissions of the report content cannot be avoided. Second, although the basic information of the patients is provided, the underlying disease status is unknown, which will involve many confounding factors and introduce uncertainty into the analysis. Third, as with other pharmacovigilance studies, the signals obtained through disproportionate analysis can only provide a correlation between drugs and hypohidrosis or anhidrosis but cannot prove the causal relationship between them, which means that the approach could be used for hypothesis generation only, not for proof. Fourth, different drugs are approved at different times, which may result in fewer adverse event reports for drugs that have been on the market for a short period.

Although these drawbacks do exist, the FAERS database can identify signals of drugs associated with hypohidrosis or anhidrosis. More importantly, this study demonstrated that a pharmacovigilance system could alternatively be used to identify anti-hyperhidrosis treatment agents and sustainably create opportunities for drug repurposing.

## Conclusion

Using pharmacovigilance for drug repurposing is an approach that is fundamentally different from all other currently employed methodologies. Based on pharmacovigilance analysis of FAERS, we have identified a plethora of candidate drugs for anti-hyperhidrosis treatment. However, more preclinical, and clinical trials are necessary to evaluate these hypotheses. Candidate drugs that were not explainable by our research should stimulate further study. The raw data and the results presented here might guide and thus accelerate these trials.

## Data Availability

The original contributions presented in the study are included in the article/supplementary material, further inquiries can be directed to the corresponding authors.
